# Successful obliteration of a wide-necked carotid terminus aneurysm with an eCLIPs flow diverting device: A case report and review of literature

**DOI:** 10.1177/2050313X261461633

**Published:** 2026-09-15

**Authors:** Alan R. Rheaume, Thomas R. Marotta, Jeremy L. Rempel, Michael M. Chow

**Affiliations:** 1 Division of Neurosurgery, University of Alberta, Edmonton, AB, Canada; 2 Department of Neurological Surgery, Stony Brook University, NY, USA; 3 Division of Diagnostic and Therapeutic Neuroradiology, Department of Medical Imaging, St. Michael’s Hospital, University of Toronto, ON, Canada; 4 Department of Radiology and Diagnostic Imaging, University of Alberta, Edmonton, AB, Canada

**Keywords:** eCLIPs device, flow diversion, intracranial aneurysm, endovascular, case report

## Abstract

Intracranial wide-neck bifurcation aneurysms are challenging to treat with conventional endovascular coiling and stenting techniques. Flow diverting devices can provide adequate neck coverage but risk branch vessel ischemia or stroke. We report the case of a 49-year-old man with an incidental wide-neck internal carotid artery terminus aneurysm, treated successfully with a stand-alone eCLIPs implant—a novel hybrid stent-like device that preserves flow across the parent arteries. At 5 months of follow-up, diagnostic angiography demonstrated complete aneurysm occlusion, and the patient remained neurologically intact. In carefully selected cases of shallow wide-neck bifurcation aneurysms, stand-alone eCLIPs device treatment may be considered as an alternative to standard flow diversion, particularly when the patency of branch or perforating vessels is paramount.

## Introduction

eCLIPs (Evasc Neurovascular Enterprises, Vancouver, BC, Canada), first described in 2008, is a novel hybrid stent-like device designed to selectively cover and flow divert across the neck of cerebral aneurysms while preserving flow in the parent arteries.^1,2^ eCLIPs is typically combined with coils in primary treatment or used secondarily to treat residual or recurrent aneurysm after intrasaccular treatments, such as coils or Woven Endobridge (WEB) devices (Terumo Neuro, MicroVention, Aliso Viejo, CA, USA).^3^ We report the first known case of internal carotid artery (ICA) bifurcation aneurysm obliteration with a stand-alone eCLIPs implant.

## Case presentation

A 49-year-old man—a non-smoker with hypertension and polycystic kidney disease (PKD)—was incidentally discovered to have an unruptured small wide-neck bifurcation aneurysm (WNBA) at the carotid terminus. He has no family history of intracranial aneurysms. He originally presented with pituitary apoplexy 3 years prior to a presumed pituitary macroadenoma that regressed and was managed conservatively. An MRA brain at this time was reported normal, but in retrospect, a shallow left ICA terminus aneurysm (4 mm neck and 1.5 mm dome) was present.

**Figure 1.**
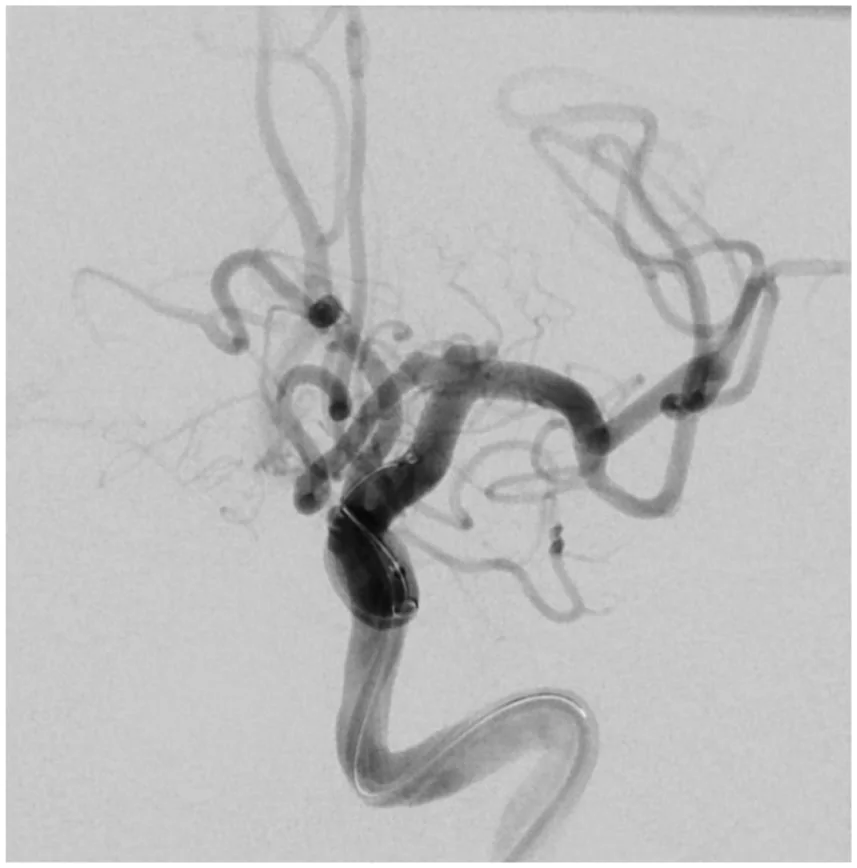
Two-dimensional digital subtraction angiography, left ICA injection (AP view) illustrating the wide-neck carotid terminus aneurysm with a small laterally-oriented lobulation. ICA: internal carotid artery.

**Figure 2.**
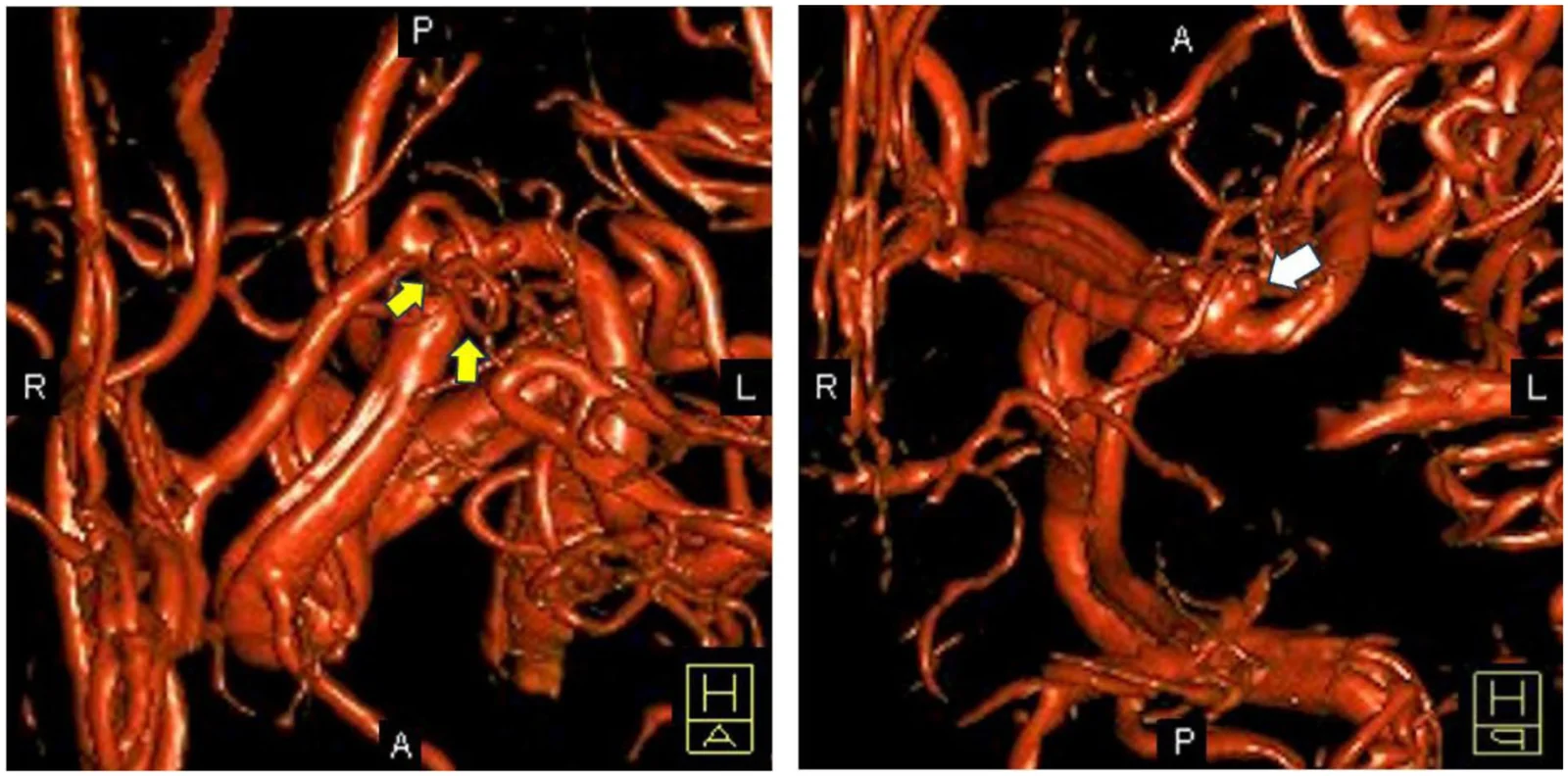
Three-dimensional rotational angiogram of the left ICA (AP views) demonstrating ICA terminus perforator branches (yellow arrows) medial to the aneurysm neck and the interval development of a small lateral aneurysm lobulation (white arrow). ICA: internal carotid artery.

Follow-up MRI sella was performed at 3 years for pituitary surveillance, along with repeat MRA brain for intracranial aneurysm screening, given his PKD. This MRA demonstrated a superiorly directed left ICA terminus WNBA with interval development of a new 2 mm laterally projecting lobulation (Figures 1 and 2). Although the size increase was modest, a multidisciplinary review between neuroradiology and neurosurgery concluded the morphological changes indicated true interval aneurysm progression, rather than measurement variability, while accounting for the spatial resolution limits of MRA. The decision to treat was based on the aneurysm progression/location, patient age/preference, and cumulative lifetime rupture risk rather than size change alone.

Clinically, the patient reported unrelated occasional occipital headaches. His neurological examination showed intact cranial nerve function, including pupillary reflexes, extraocular movements, and visual fields, preserved visual acuity, and no focal deficits. Ophthalmologic evaluation did not detect visual field defects, papilledema, or fundoscopic abnormalities.

Elective endovascular treatment was undertaken using an eCLIPs device. Treatment alternatives included: (i) balloon-assisted or stent-assisted coiling, although this would have been higher risk due to the broad, short, and multilobulated aneurysm morphology; (ii) a traditional flow diverter (FD), although this might have inadvertently sacrificed the ipsilateral A1; and (iii) surgical clipping. Dual-antiplatelet therapy with aspirin and clopidogrel was started 1 week pre-procedure.

## Neuroendovascular procedure and outcome

A BMX96 guide (Penumbra, Alameda, CA, USA), Vecta 74 intermediate (Stryker, Kalamazoo, MI, USA), and Headway 27 microcatheter (Terumo Neuro, MicroVention) system was used. The left A1 was selected, and a second-generation 3.25 × 6 mm eCLIPS flow diverting stent device was deployed between the left A1 segment into the carotid terminus, then advanced across the aneurysm neck into the left M1 by advancing the microcatheter into the left M1.

**Figure 3.**
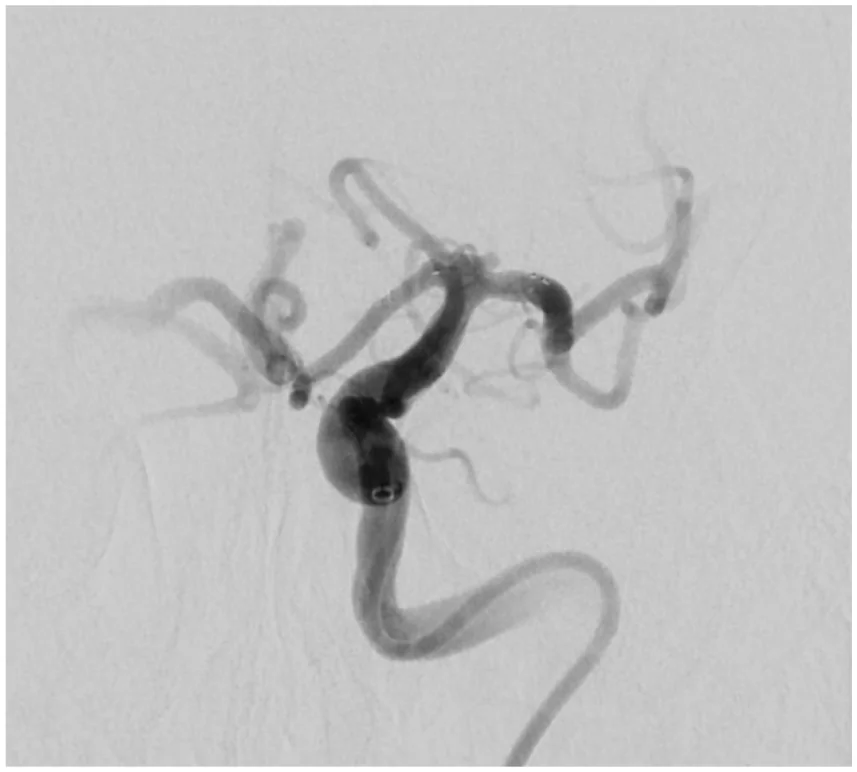
Left ICA injection (AP view) demonstrating the final intraoperative position of the eCLIPs device post-deployment. ICA: internal carotid artery.

Control angiography showed satisfactory device positioning post-deployment and significant contrast stasis within the aneurysm, consistent with an O’Kelly–Marotta (OKM) B3 score (Figure 3).^4^

Post-procedure, the patient was neurologically intact. Early postoperative diffusion-weighted MRI was not obtained as the patient remained neurologically asymptomatic. He was discharged home on postoperative day 1 and remained well at 6-month follow-up. Dual-antiplatelet therapy continued for 3 months, followed by lifelong daily aspirin monotherapy.

## Follow-up imaging

**Figure 4.**
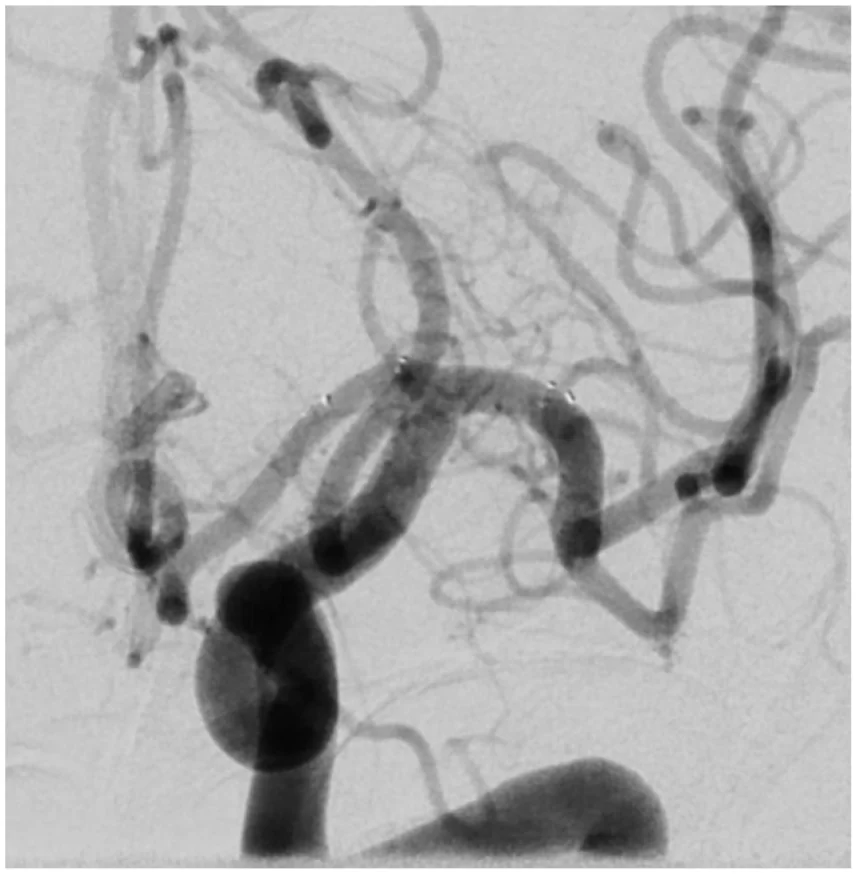
Left ICA injection (AP view) showing complete ICA aneurysm obliteration and vessel patency at 5 months follow-up post-eCLIPs device treatment. ICA: internal carotid artery.

A 5-month post-procedure follow-up angiogram showed no significant residual filling of the ICA terminus aneurysm, for an OKM-D3 and modified Raymond–Roy Occlusion Class I outcome (Figure 4).^4,5^ There was a questionable, very delayed filling in the aneurysm neck region seen only on magnified AP films. The left ICA, A1, and M1 were widely patent with good apposition of the eClips device and no neurovascular complications (Supplemental Material).

## Discussion

We report the first known case of complete shallow WNBA obliteration with an eCLIPs device without adjunctive coils or intrasaccular flow disruption. Unlike conventional FDs, eCLIPs is designed to flow divert across the aneurysm neck while minimizing metal coverage across perforator-rich parent vessels.^6^ The rationale for stand-alone eCLIPs in this case was the shallow, broad-based aneurysm morphology and the perforation risk while catheterizing such an aneurysm. A key advantage of this treatment strategy was avoidance of branch vessel jailing associated with conventional FD placement, which may increase the risk of perforator ischemia or inadvertent vessel sacrifice, although this risk appears low in supratentorial vessels.^7,8^

eCLIPs is less constrained by WNBA dimensions or morphology than intrasaccular devices, and can treat aneurysms with extremely low dome-to-neck ratios.^6^ In the present case, only conventional FD was a suitable endovascular alternative due to the shallow aneurysm morphology. Although FD for WNBAs is generally well tolerated, anatomic suitability must balance the risks of perforator ischemia, vessel occlusion, and stroke associated with jailed branch vessels. Recently, surface-modified (SM) “coated” FDs have emerged as a potential strategy to decrease platelet adhesion and thrombogenicity in an effort to reduce FD ischemic complications.^9^ Although early results suggest comparable results to “uncoated” FDs, multiple trials are ongoing, and the role of SM FDs in WNBAs requires further definition.^9^

eCLIPs has demonstrated favorable technical and angiographic outcomes for both primary and recurrent bifurcation aneurysm treatments.^1,2,6,10,11^ A meta-analysis of 110 WNBAs treated with eCLIPs and coils demonstrated successful implantation in 93%, with 61% complete/near complete occlusion at follow-up.^10^ These occlusion rates are similar to FD for bifurcation aneurysms (68% complete occlusion at follow-up),^12^ yet lower than Y-stent-assisted coiling (82.2% immediate near/complete occlusion, 95.4% at follow-up).^13^ In the recent French multicenter prospective EESIS-FR trial of 123 carotid or basilar terminus aneurysms, eCLIPs achieved 93% implantation success, although long-term and angiographic outcomes remain pending.^11^

Safety outcomes with eCLIPs appear comparable to alternative WNBA treatments. EESIS-FR reported low rates of primary safety events (2.4%), minor stroke (4.1%), and thromboembolic complications (3.7%), compared to 2.5%–14.4% thromboembolic complications reported for WEB,^3,14^ Contour (Stryker, Fremont, CA, USA),^14^ and ARTISSE (Medtronic, Irvine, CA, USA).^15^ Mehrizi et al. reported low rates of ischemic stroke (0%–5%) and treatment-mortality (0%–4%) associated with eCLIPs.^10^ Similar complication profiles have been reported for Y-stent-assisted coiling (ischemia 7%, morbidity 2.4%, mortality 1.1%).^10^ In contrast, FD treatment of bifurcation aneurysms has been associated with higher ischemic/thromboembolic events (16%), including 7% permanent symptoms, most commonly attributed to jailed artery stasis or in-stent thrombosis.^11^ However, cross-study comparisons remain limited by variable inclusion of asymptomatic ischemic or perforator-related complications, ruptured versus unruptured aneurysms, differing WNBA locations, and self-adjudicated outcomes subject to reporting bias.

Overall, current evidence suggests eCLIPs provides comparable technical success, safety, and efficacy of eCLIPs for WNBAs, although available evidence is limited by heterogeneous study design, safety definitions, follow-up durations, and outcome reporting. Ultimately, WNBA treatment selection must balance the likelihood of durable occlusion against risks of rupture, thromboembolism, branch or perforator compromise, ischemic stroke, and recurrence.

This report is limited to a single case, and further studies are required in larger, prospective cohorts to characterize the long-term safety, efficacy, and durability of eCLIPs for WNBAs. Finally, a diffusion-weighted MRI was not performed in this asymptomatic patient, although standardized postoperative MRI protocols may identify silent perforator ischemic lesions to further define the eCLIPs safety profile.

## Conclusion

In carefully selected shallow WNBAs, stand-alone eCLIPs implantation may be considered as a treatment alternative to standard flow diversion, particularly when branch or perforator preservation is paramount. Future studies are required to validate eCLIPs comparative safety, efficacy, durability, and long-term outcomes.

## Supplemental Material

sj-docx-1-sco-10.1177_2050313X261461633 – Supplemental material for Successful obliteration of a wide-necked carotid terminus aneurysm with an eCLIPs flow diverting device: A case report and review of literature Supplemental material, sj-docx-1-sco-10.1177_2050313X261461633 for Successful obliteration of a wide-necked carotid terminus aneurysm with an eCLIPs flow diverting device: A case report and review of literature by Alan R. Rheaume, Thomas R. Marotta, Jeremy L. Rempel and Michael M. Chow in SAGE Open Medical Case Reports

## References

[1] Diestro JD Keough MB Ashforth RA , et al. Treatment of wide-necked bifurcation aneurysms with the eCLIPs device: 5-year experience of a single center. Journal of Neurointerv Surg 2023; 15(5): 461–464. 35545426 10.1136/neurintsurg-2022-018731

[2] Consoli A Nishi H Diouf A , et al. Endovascular treatment of wide-neck bifurcation aneurysms: the eCLIPs device. J Neurointerv Surg 2024; 16(3): 229. 38171614 10.1136/jnis-2023-020442

[3] Pierot L Moret J Barreau X , et al. Safety and efficacy of aneurysm treatment with WEB in the cumulative population of three prospective, multicenter series. J Neurointerv Surg 2018; 10(6): 553–559. 28965106 10.1136/neurintsurg-2017-013448 PMC5969386

[4] O’Kelly CJ Krings T Fiorella D , et al. A novel grading scale for the angiographic assessment of intracranial aneurysms treated using flow diverting stents. Interv Neuroradiol 2010; 16(2): 133–137. 20642887 10.1177/159101991001600204 PMC3277972

[5] Mascitelli JR Moyle H Oermann EK , et al. An update to the Raymond–Roy occlusion classification of intracranial aneurysms treated with coil embolization. J Neurointerv Surg 2015; 7(7): 496–502. 24898735 10.1136/neurintsurg-2014-011258

[6] De Vries J Boogaarts HD Sørensen L , et al. eCLIPs bifurcation remodeling system for treatment of wide neck bifurcation aneurysms with extremely low dome-to-neck and aspect ratios: a multicenter experience. J Neurointerv Surg 2021; 13(5): 438–442. 32788388 10.1136/neurintsurg-2020-016354 PMC8053345

[7] Cagnazzo F Lefevre PH Mantilla D , et al. Patency of the supraclinoid internal carotid artery branches after flow diversion treatment. A meta-analysis. J Neuroradiol 2019; 46(1): 9–14. 30099016 10.1016/j.neurad.2018.07.006

[8] Gawlitza M Januel AC Tall P , et al. Flow diversion treatment of complex bifurcation aneurysms beyond the circle of Willis: a single-center series with special emphasis on covered cortical branches and perforating arteries. J Neurointerv Surg 2016; 8(5): 481–487. 25878068 10.1136/neurintsurg-2015-011682

[9] Ma L Hoz SS Al-Bayati AR , et al. Flow diverters with surface modification in patients with intracranial aneurysms: a systematic review and meta-analysis. World Neurosurg 2024; 185: 320–326. 38160909 10.1016/j.wneu.2023.12.132

[10] Mehrizi MA Habibi MA Keykhosravi E , et al. The safety and efficacy of eCLIPs for treatment of wide-necked bifurcation aneurysms: a systematic review and meta-analysis. World Neurosurg 2023; 180: 213–223. 37813336 10.1016/j.wneu.2023.10.011

[11] Blanc R Riva R Levrier O , et al. eCLIPs, a dedicated neck bridging device for wide neck bifurcation aneurysms: procedural and 30 day safety results of the French prospective trial. J Neurointerv Surg 2026; 2026: 1–7. 10.1136/jnis-2025-024503 41813106

[12] Kashkoush A El-Abtah ME Petitt JC , et al. Flow diversion for the treatment of intracranial bifurcation aneurysms: a systematic review and meta-analysis. J Neurointerv Surg 2024; 16(9): 921–927. 37541838 10.1136/jnis-2023-020582

[13] Cagnazzo F Limbucci N Nappini S , et al. Y-stent-assisted coiling of wide-neck bifurcation intracranial aneurysms: a meta-analysis. Am J Neuroradiol 2019; 40(1): 122–128. 30523146 10.3174/ajnr.A5900 PMC7048580

[14] Hecker C Broussalis E Pfaff JA , et al. Comparison of the Contour Neurovascular System and Woven EndoBridge device for treatment of wide-necked cerebral aneurysms at a bifurcation or sidewall. J Neurosurg 2023; 139(2): 563–572. 36708532 10.3171/2022.12.JNS222268

[15] Hohenstatt S Costalat V Dargazanli C , et al. New ARTISSE intrasaccular device for intracranial aneurysm treatment: short term clinical and angiographic result from the prospective registry INSPIRE-A. J Neurointerv Surg 2026; 18(2): 558–567. 39880621 10.1136/jnis-2024-022576 PMC12911556

