# Supplementary material for: Successful obliteration of a wide-necked carotid terminus aneurysm with an eCLIPs flow diverting device: A case report and review of literature
Source: SAGE Open Med Case Rep. 2026 Sep 15;14:2050313X261461633. doi: 10.1177/2050313X261461633 (PMC13583283; doi:10.1177/2050313X261461633)
Supplement: Supplementary file 1 [file sj-docx-1-sco-10.1177_2050313X261461633.docx]

**SUPPLEMENTAL MATERIAL:**

***Supplemental Figure 1:*** *schematic of an eCLIPs device (Evasc Medical Systems Corp., Vancouver, British Columbia, Canada), which features a flow-diverting leaf segment spanning the aneurysm neck and anchor segment distally within a branch vessel, with proximal, centre, and distal fluoroscopic markers demarcating each segment.*

**Source:** Evasc Neurovascular Enterprises. eCLIPs Products [Internet]. Vancouver, Canada. 2025 [cited 2025 June 24]. Available from: https://www.evasc.com/features.
